# Analysis of the effect of metal ions on the ability of Xylanase to hydrolyze wheat bran by molecular dynamics simulations

**DOI:** 10.3389/fbioe.2023.1142873

**Published:** 2023-02-16

**Authors:** Mingrui Hou, Chuanqi Liang, Yanan Fei, Dan Yang, Ningjing Zhang, Yujie Lu, Lei Wang, Zhiqiang Xing, Zongpei Zhao

**Affiliations:** ^1^ School of Grain Science and Technology, Jiangsu University of Science and Technology, Zhenjiang, China; ^2^ Jiahe Foods Industry Co., Ltd., Suzhou, China

**Keywords:** xylanase, immobilization, metal ion, molecular dynamic (MD) analysis, activity

## Abstract

**Introduction:** Wheat bran is the main by-product of wheat processing, containing about 30% pentosan and 0.4%–0.7% ferulic acid. Wheat bran is the main raw material used to prepare feruloyl oligosaccharides by hydrolysis of Xylanase, we discovered that the ability of Xylanase to hydrolyze wheat bran could be affected in the presence of different metal ions.

**Methods:** In the present study, we have probed the effects of different metal ions on the hydrolysis activity of Xylanase on wheat bran and tried to analyze the effect of Mn^2+^ and Xylanase by molecular dynamic (MD) simulation.

**Results:** Our results suggested that Mn^2+^ had improved the Xylanase hydrolyzing wheat bran to obtain feruloyl oligosaccharides. Particularly when the concentration of Mn^2+^ reached 4 mmol/L, the optimal product has been obtained 2.8 times higher to compare with no addition. Through the MD simulation analysis, our results reveal that Mn^2+^ can induce structural change in the active site, which enlarges the substrate binding pocket. The simulation results also revealed that the addition of Mn^2+^ resulted in a low RMSD value compared with the absence of Mn^2+^ and helped stabilize the complex.

**Conclusion:** Mn^2+^ could increase the enzymatic activity of Xylanase in the hydrolysis of feruloyl oligosaccharides in wheat bran. The finding could have significant implications for the preparation of feruloyl oligosaccharides from wheat bran.

## 1 Introduction

Metal ions play an important role in cell mechanisms, including nucleic acid and protein structure stabilization, enzyme catalysis, signal transduction, muscle contraction, photosynthesis, and respiration. They are the simplest but most versatile cofactors in protein biochemistry (Dudev and Lim, 2014). Among the metal cations, K^+^, Ca^2+^, Zn^2+^, Mg^2+^, Cu^2+^, and Mn^2+^ are in various biochemical processes which could help improve the enzyme activity (Bertini and Sigel, 2001). The regulation of enzymes through metal ions is widespread in biology and emphasizes a physiological need for stability and high catalytic activity (Gohara and Di Cera, 2016; Bloom and Lancaster, 2018). Many studies also have revealed that metal ions could influence the activity of Xylanase, such as thermostable xylanase that Ca^2+^, Pb^2+^, K^+^, Mn^2+^, and Cu2+ could enhance enzyme activity from 6.4% to 29.9% endo-xylanase from *Thermoanaerobacterium bryantii strain mel9* ^*T*^ that Mn^2+^ could improve the activity of the enzyme about 10%–25%, 1,4-β-D-xylanase from wheat straw (Ca^2+^ could improve the activity about 27%) (Ghanem et al., 2000; Basit et al., 2020; Yi et al., 2022). However, according to our knowledge there is still no further studies could reveal more information on the effect of metal ions on xylanase from wheat bran by using MD.

As the main by-product of wheat processing, wheat bran is rich in cellulose and hemicellulose (Klepacka and Fornal, 2006). Oligosaccharides can be produced by hydrolysis of hemicellulose in wheat bran by enzymatic hydrolysis (Wang and Lu, 2013). Ferulic acid can be obtained by hydrolysis of oligosaccharides with the use of specific enzymes such as Xylanase from fungi, bacteria, and yeast (Ou and Kwok, 2004). Ferulic acid is one of the ubiquitous compounds in nature, especially in the form of esters rich in plant cell walls (Kikuzaki et al., 2002). Ferulic acid as an antioxidant is not only currently considered to prevent the oxidation of lipids in food but also to prevent free radical-induced diseases such as cancer, atherosclerosis, and ageing caused by oxidative tissue degeneration (Wheeler, 2003).

The structural diversity of Xylanase requires enzymes that cleave internal glycosidic linkages (depolymerizing action) and enzymes that break the spatial obstacles (branch points) that limit the formation of the enzyme-substrate complex in the polysaccharide backbone (Moreira, 2016). And Endo-Xylanases can catalyze the random cleavage of β-1,4-linked. D-xylopyranose units from the homopolymer backbone structure of xylose, and then the wheat bran was hydrolyzed by endo-Xylanase to generate xylo-oligosaccharides (XOS), followed by the release of feruloyl oligosaccharides (Babbar and Oberoi, 2014). When different types and concentrations of metal ions act on the activity of Xylanase it will affect the yield of feruloyl oligosaccharides (Deng et al., 2019).

However, the mechanism and influence of different metal ions on Xylanase have not been fully understood. In order to investigate the role of metal ions in the hydrolysis reaction, the molecular dynamics simulation could provide a different angle of the view. By using the molecular dynamics method, we could obtain the dynamic information between enzyme and ligand such as the simulated result of Root Mean Square Deviation (RMSD), and the flexibility of critical residues of the Xylanase by the Root Mean Square Fluctuation (RMSF).

In the present study, Xylanase was expressed and purified by *Escherichia coli* from a previous report (Huang et al., 2021), and it was used to enzymatically hydrolyze the wheat bran to prepare feruloyl oligosaccharides. The effect of metal ions on the preparation of feruloyl oligosaccharides through hydrolyzing wheat bran by Xylanase was studied. Molecular dynamics simulations were used to analyze the role of the metal ion (Weiner et al., 1984).

## 2 Materials and methods

### 2.1 Wheat bran

Before washing the wheat bran in water, the large straw part had been removed. Weigh 15 g of wheat brans that have been refined and measured the moisture value, which was 3.47%. Then the brans were repeatedly washed with water until the washout is clarified. This method could help remove some of the soluble starch from the surface of the bran. After the cleaning is completed, it was poured into trays and put into an oven at 105°C for 4 h. The specific drying time depends on the moisture content of the sample, and when the moisture content reaches about 0%, it can be regarded as completely dried, and the sample can be crushed by using a high-speed crusher (Dongmai DM-500G, China).

Add 60 mL of Distilled De-Ionized (DDI) to the pretreated wheat bran sample and steamed it in a water bath for 30 min. After steaming, adjusted the pH of the solution to 5.6 using 0.1 mol/L HCl, add 1.5% of diluted high-temperature resistant α-amylase, and reacted in a water bath at 65°C for about 3 h. During the enzymatic digestion, stir the solution from time to time using a glass rod to ensure that the amylase and wheat bran was fully contacted. The enzymatic digestion time took 3 h, and iodine solution was added to the solution.

After digestion, the pH was adjusted to 9 by using 1 mol/L NaOH and added 1% alkaline protease. After the reaction at 65°C for 4 h. The residual enzyme was inactivated by boiling water for 10 min. Centrifuged at 5000 r/min for 20 min, poured off the supernatant, rinsed the precipitate several times with hot distilled water until the washing solution was not turbid, then used 70% ethanol and 1% NaOH solution, respectively for washing two times, centrifuged at 5,000 r/min for 20 min, the precipitate was treated with the freeze-drying method in order to obtain insoluble dietary fiber with starch.

The insoluble dietary fiber after freeze-drying was weighed and the average insoluble dietary fiber mass was 2.5567 g. It accounted for 51% of the original wheat bran.

### 2.2 Scanning electron microscopy

The insoluble dietary fibers obtained from the preparation were observed by scanning electron microscopy. And images were taken under different magnifications of observation.

### 2.3 Gene expression of Xylanase

The gene encoding the Xylanase was synthesized according to the previously reported sequence (Cheng et al., 2014). The fragment of Xylanase was amplified using forward primer (5′-AAA​AAC​ATA​TGC​AGA​GCT​TTT​GTA​G-3′) and reverse primer (5′-TTT​TTC​TCG​AGT​TAA​TCG​CCA​ATG​T-3′). We performed PCR amplifications under conditions of 94°C incubation for 5 min followed by 35 cycles of 94°C for 1 min, followed by 30 s at 55°C 1 min at 72°C, and a final 10 min of incubation. There were two restriction enzyme sites (NdeI and XhoI) were used for cloning the target gene into the pET-28a (+) expression vector, and *E. coli* BL21 strains were used for the expression.

### 2.4 The effect of metal ions on the activity of Xylanase

In order to test the hydrolysis ability, six different Xylanase gradient has been prepared: 0.1 g of the insoluble dietary fiber were tested with 0, 0.5, 1, 1.5, 2, 2.5 mL Xylanase with a final volume of 5 mL. Incubated at 40°C for 4 h and boiled for 15 min for inactivation. Centrifugation was carried out at 3,500 × g for 10 min. Then, 0.1 mL of the supernatant from centrifugation mixed with 0.9 mL of 0.1 M boric acid-glycine buffer at pH 10 and measured the absorbance.

The metal ions (Na^+^, Zn^2+^, Ca^2+^, K^+^, Cu^2+^, Mg^2+^, and Mn^2+^) were prepared with following concentration orders: 4, 6, 8, and 10 mM. All chemicals and solvents used are analytical grade and purchased from Aladdin Ltd. (Shanghai, China). One unit of Xylanase is defined as the amount of enzyme consumed to release 1 μmol of xylose reducing substances per minute.

### 2.5 Molecular dynamics simulation

In order to get more information from the structure view, three systems for Molecular dynamics (MD) simulations have been set: free, Xylanase-xylose complex with additional Mn^2+^ with two different concentrations (4 and 6 mM). The simulations were performed by using the OPLS-AA force field and GROMACS 2020.1 (https://www.gromacs.org/) simulation package. All complexes were individually placed into a water-filled cubic box at a distance of 10 Å from the edge of the molecule. In the presence of two different concentrations of Mn^2+^ (4 and 6 mM), 14,801 water molecules were filled separately. For the free complex, three Na^+^ were added to the system in order to achieve an electro-neutrality system. We set different numbers of ions to vary the concentration of Mn^2+^ in the system. One Mn^2+^ and two Mn^2+^ were added to the systems separately that equal to 4 and 6 mM/L and the system has been neutralized. The starting structure of Xylanase was derived from PDB (PDB code: 3WP4) and the topology file of the ligand was prepared by using LigParGen (http://zarbi.chem.yale.edu/ligpargen/index.html). Each system was equilibrated by 100 ps MD simulation in the canonical (NVT) ensemble and 100 ps MD simulation in the isothermal–isobaric (NPT) ensemble using position restraints on the heavy atoms of the protein to allow for the equilibration of the solvent (Gheibi et al., 2019). And the system pressure was maintained constant at 1 atm by the Parrinello-Rahman coupling method. The electrostatic interactions were calculated by using the Particle Mesh Ewald (PME) method with 1.0 nm short-range electrostatic and van der Waals cutoffs. The temperature of equilibration was set at 300 K. All systems carried out the MD simulations for 100 ns and the time step of the MD simulations is 2 fs. The non-bonded cutoff was set to 10 Å. The non-bonded pairs are updated every 10 steps. Furthermore, structural stability is measured by the RMSD (Root Mean Square Deviation) calculated by superimposing the atoms’ starting structures in the MD (Bjelkmar et al., 2010). In the end, the RMSD, the Root Mean Square Fluctuation (RMSF), the Radius of Gyration (R_g_), and Hydrogen-bond numbers were calculated and plotted by SigmaPlot. The catalytic pocket was predicted by the POCASA (https://altair.sci.hokudai.ac.jp/g6/service/pocasa/) (Senes et al., 2001; Hong and Lei, 2009; Yu et al., 2010).

## 3 Results

### 3.1 Scanning electron microscopy analysis

**FIGURE 1:**
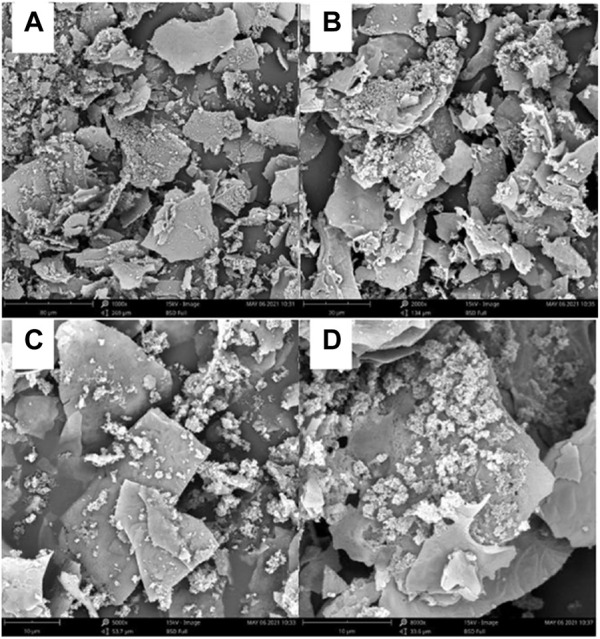
The prepared insoluble dietary fiber under an electron microscope. **(A, B)** were observed under magnifications at 1,000 × and 2000 × , respectively. **(C, D)** were observed under magnifications at 5,000 × and 8,000 × .

After scanning electron microscopy (the images were photographed after pre-processing), it was observed that the insoluble dietary fiber was basically in the form of irregular fragments, and the fragments also contained some fibrous material (Figure 1).

### 3.2 The hydrolysis activity of Xylanase on the wheat bran

**FIGURE 2:**
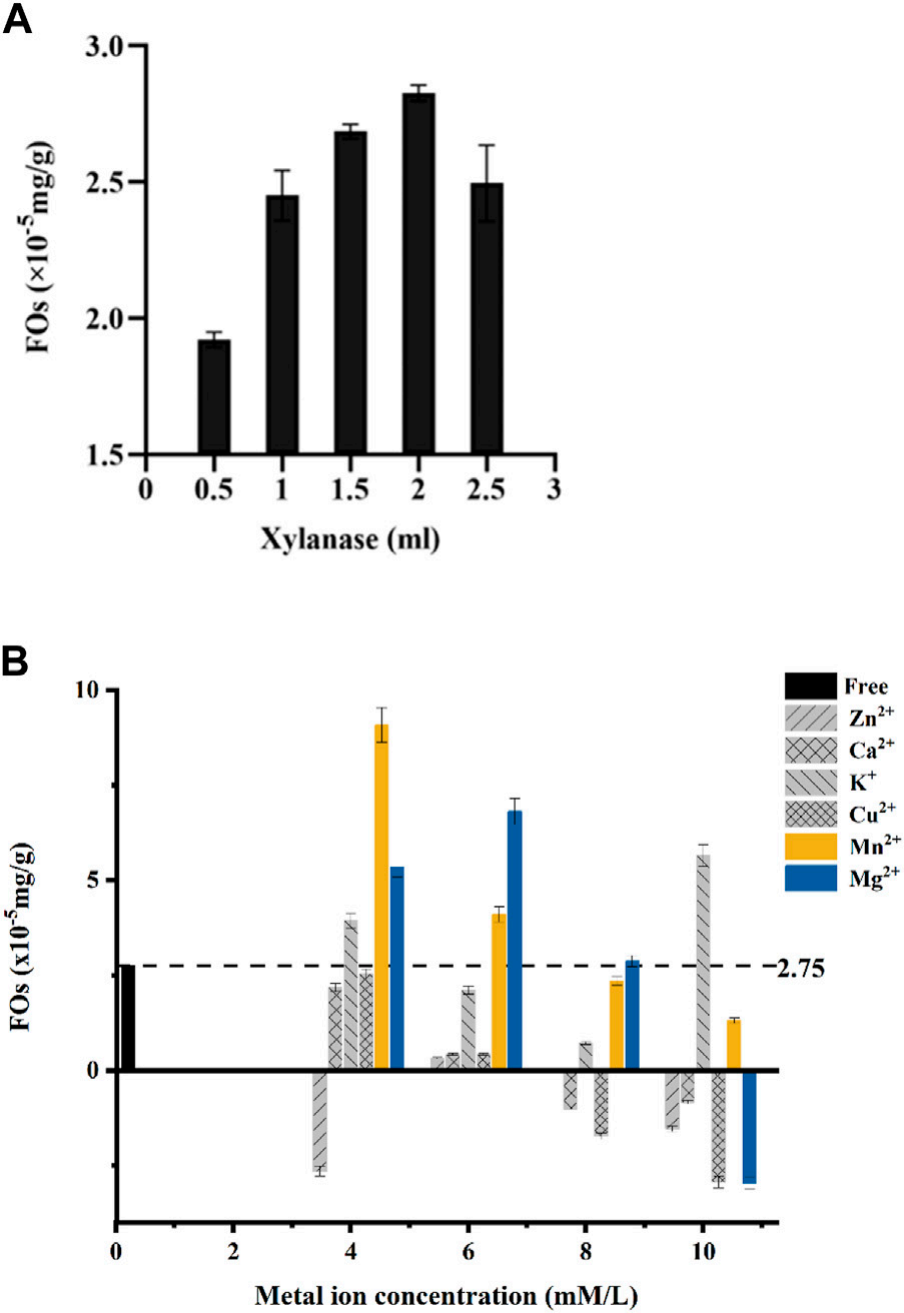
**(A)** Xylanase solution amounts to the preparation of ferulic acid (FOs) esters by Xylanase hydrolysis of wheat bran. **(B)** Concentrations of metal ions in the preparation of ferulic acid (FOs) esters by hydrolysis of wheat bran by Xylanase.

Our results showed that added 2 mLXylanase (100 U) mixed with 0.1 g dietary fiber could obtain the highest feruloyl oligosaccharides. When more than 2 mL of Xylanase was added in the reaction oligosaccharides production would have a sharp drop (Figure 2A). The decreasing production could be due to the enzymatic hydrolysis in the ester bond of feruloyl oligosaccharides which the released of the free ferulic acid and inhibited the activity.

### 3.3 The role of metal ions in Xylanase enzyme activity

Meanwhile, the production of feruloyl oligosaccharides was closely related to the concentration of metal ions in the solution from our study. Most of the metal ions (Ca^2+^, K^+^, Mg^2+^, and Mn^2+^) could improve the activity at 4–6 mM concentrations (Figure 2B).

Through gradient experiments, our results showed a significant correlation between ion concentration and feruloyl oligosaccharide production. At ion concentrations equal to 4 mM, only Zn^2+^ showed an inhibitory effect, and all other metal ions increased Xylanase activity and feruloyl oligosaccharide production. While at 6 mM, all the ions showed a promoting effect on Xylanase activity, Mg^2+^ was increased relative to 4 mM and the K^+^, Ca^2+^, Cu^2+^, and Mn^2+^ showed a decreasing trend. With the further increase of metal ion concentration of Ca^2+^, Cu^2+^, K^+^, Mg^2+^, and Zn^2+^ which began to show an inhibitory effect on the Xylanase activity. Cu^2+^ inhibiting highest, while Zn^2+^ and Ca^2+^ inhibited less. In the concentration range of 4–10 mM, the promoting effect of Mn^2+^ on Xylanase decreased with increasing concentration (Figure 2B).

In our gradient experiments, 4 mM Mn^2+^ could improve the hydrolysis of Xylanase ability and result in 2.8 times higher feruloyl oligosaccharides yield from wheat bran than no addition Mn^2+^. We also discovered that the optimum concentration of Mn^2+^ was 4 mM when wheat bran could release feruloyl oligosaccharides more efficiently.

### 3.4 Dynamics simulation

#### 3.4.1 Root mean square deviation

**FIGURE 3:**
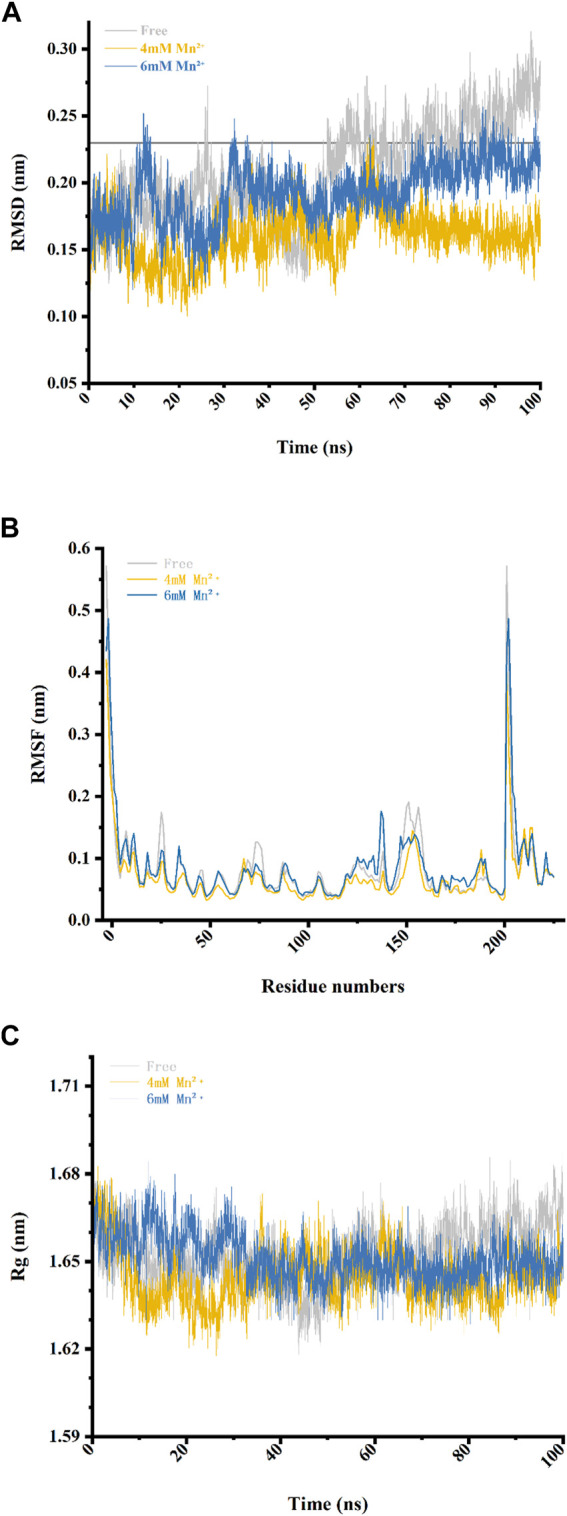
Four data analysis plots of the free complex (the Xylanase complexed with xylose in solvent) and complex-Mn^2+^(the Xylanase complexed with xylose in 4 and 6 mM of Mn^2^⁺) during the 100 ns MD simulations. **(A)** RMSD values during the 100 ns MD simulations. **(B)** RMSF values of residues during the 100 ns MD simulations. **(C)** The radius of gyration (R_g_) values of the three complexes.

In order to investigate the stability of the Xylanase-xylose complex structure, we calculated its RMSD by superimposing the starting structure of atoms to measure its stability in MD simulation (Table 1). As shown in Figure 3A, the overall RMSD fluctuation of the free Xylanase-xylose complex is larger and more unstable throughout the MD simulation compared to the simulation with Mn^2+^. The fluctuation of RMSD values of the Xylanase-xylose complex in the period of 60–100 ns was around 0.15–0.23 nm in the two cases of Mn^2+^ concentration of 4 mM and 6 mM, respectively. And the deviations of RMSD values in this range are small and the complexes maintain a steady state throughout this phase of the simulation, indicating that the complexes are more stable under the influence of metal ions. In the last 40 ns of the simulation, the RMSD values of the complexes were basically below 0.23 nm, while most of the RMSD values of the system without Mn^2+^ ions were >0.23 nm (Figure 3A), which indicated that the addition of Mn^2+^ helps the complexes more stable. As shown in Figure 3A, Mn^2+^ showed more stable results with the complex structure at a concentration of 4 mM than 6 mM, which is consistent with the results obtained in Figure 2B that different concentrations of metal ions have different catalytic effects on Xylanase activity. The results of RMSD suggested that Mn^2+^ has been proven that could improve catalytic activity better at 4 mM.

**TABLE 1:** The RMSD and RMSF and the standard deviation average values in free complex and complexes with Mn^2+^ in different concentrations.

Simulated system	RMSD (nm)	RMSF (nm)
Free	0.207 (±3.54E^−2^)	0.0771 (±5.27E^−2^)
4 mM Mn^2^⁺	0.186 ± 2.12E^−2^)	0.0727 (±4.04E^−2^)
6 mM Mn^2^⁺	0.193 (±2.19E^−2^)	0.0784 (±5.06E^−2^)

#### 3.4.2 Root mean square fluctuation

To reveal direct insights into the flexibility and structural fluctuations of complexes, their RMSF values of them in different concentrations of Mn^2+^ were calculated. The RMSF for all MD simulations has slight fluctuation and most of the fluctuation is range from 0.04 to 0.19 and did not exceed 0.20 nm (Table 1). It is worth noting that in Loop 1 (Gly-11 to Ala-40), Loop 2 (Arg-60 to Asp-80) and Loop 3 (Pro-120 to Gln-160) RMSF values in the free complex are higher than those Loops with Mn^2+^ involved. The fluctuations of the three loops may reveal that the region of the protein is involved in interaction with metal ions in the Xylanase-xylose complex. As shown in Figure 3B, the complex in 4 mM Mn^2+^ has the lowest value which can be referred to as that at this concentration the complex is more stable. It also suggested that the presence of Mn^2+^ in the system could interact with these loops and could lead to conformation changes in the Xylanase.

**FIGURE 4:**
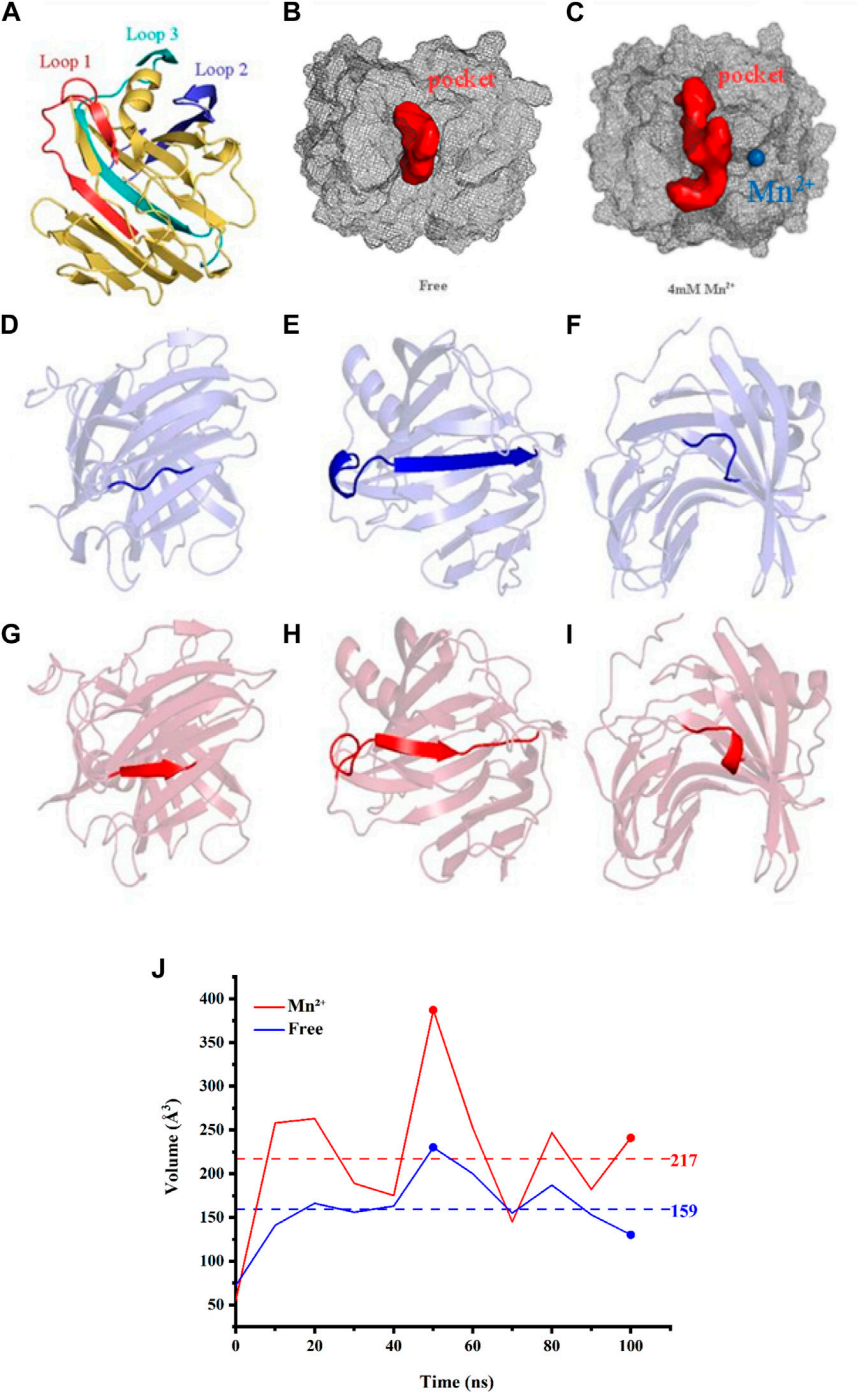
**(A)** The catalytic residues are presented in the form of stick structures. Loop 1 (Arg-60 to Ala-80) is illustrated in a red cartoon while Loop 2 (His-130 to Gln-160) is in deep blue. Loop 3 (Lys-185 to Ser-210) is in cyan. Active pockets are indicated by pale cyan. **(B)** The binding pockets of free complex (red balls). **(C)** The binding pockets of the complex (red balls) with Mn^2+^ (blue ball). **(D)** The common structure of free complex in Loop1. **(E)** The combined structure of α-helix and β-sheet of the free complex in Loop1. **(F)** The common structure of free complex in Loop2. **(G)** The β-sheet of the complex with Mn^2+^ in Loop 1. **(H)** The β-sheet of the complex with Mn^2+^ in Loop1. **(I)** The α-helix of the complex with Mn^2+^ in Loop2. **(J)** The volumes change of the binding pockets of three tested Xylanases, as a function of time.

In accordance with the analysis of POCASA, Loop 1, 2, and 3 are all located in the catalytic pocket (Figures 4A–C). The results indicated that the conformation change of Loop 1, 2, and 3 may enlarge the entrance to the catalytic pocket from 55 Å³ to 387 Å³, rendering the active site accessible to substrates more easily. And the change in pocket volume is also shown in Figure 4J, and Table 2.

**TABLE 2:** The change of pocket volume of the Xylanase-xylose complex in the whole simulation.

Time (ns)	0	10	20	30	40	50	60	70	80	90	100
Volume (Å^3^)	55	258	263	189	175	387	252	145	247	182	241

Therefore, the presence of Mn^2+^ does have an effect on the binding pocket of Xylanase and can cause conformation changes.

#### 3.4.3 Radius of gyration

Through the compactness of a protein was indicated by describing the radius of gyration (Rg) could suggested that proteins having the lowest radius of gyration, have a more compact structure. Therefore, proteins that had a less radius of gyration were more stable. In Figure 3C and Table 3, with the increase of metal ions’ concentration, the radius of gyration was reduced, which indicated that more compactness and more rate of folding of the protein taken place in the complex mixed with Mn^2+^ compared with the free complex. However, when the concentrations of metal ions became higher, a completely opposite effect would happen. This was consistent with the results of Figure 2B.

**TABLE 3:** The mean of the radius of gyration and the standard deviation average values in free complex and complexes with Mn^2+^ in different concentrations.

Simulated system	Rg (nm)
Free	1.65 (±9.73E^−3^)
4 mM Mn^2^⁺	1.64 (±8.96E^−3^)
6 mM Mn^2^⁺	1.65 (±7.91E^−3^)

#### 3.4.4 Hydrogen-bond numbers

The hydrogen-bond numbers are one of the most vital analyses to evaluate the stability of a protein. As depicted in Table 4, it indicates that the presence of Mn^2+^ results in the number of hydrogen bonds in the protein increasing. The highest hydrogen bond numbers take place in 4 mM of Mn^2+^. The increase of Hydrogen-bond numbers in presence of 4 mM Mn^2+^ indicates a stronger correlation and higher stability of the complex in this simulation. However, with more ions added, the reverse action occurred. It suggested that the stability of the protein would decrease with the concentration increased.

**TABLE 4:** The number of hydrogen bonds and the standard deviation average values in free complex and complexes with Mn^2+^ in different concentrations.

Simulated system	Free	4 mM Mn^2^⁺	6 mM Mn^2^⁺
Number of Hydrogen bonds	174 (±7.00)	176 (±5.71)	175 (±6.03)

## 4 Discussion

In the present study, the mechanism and effect of different metal ions on Xylanase are not fully understood. In order to analyze the role of metal ions in the hydrolysis reaction, we investigated the capacity of different metal ions catalyzing Xylanase to hydrolyze wheat bran at different concentrations in our experiments. The results showed that the catalytic effect was generally better among the six tested metal ions at a Xylanase concentration of 2 mL and a metal ion concentration of 4 mM Mn^2+^ showed a better improvement for catalyzing Xylanase’s hydrolysis of wheat bran. For further verification, we chose to perform molecular dynamics simulations to study further. The simulation findings demonstrated that when Xylanase was in the Mn^2+^ solution environment, its conformation altered while it was not in water. The analysis of the simulated trajectory graphs derived for Xylanase showed that the binding pocket of Xylanase enlarged as the simulation went on, which may be due to the presence of Mn^2+^ causing conformation changes in the protein and thus affecting the binding pocket. In a further study, we found that there are huge conformation changes at the residues which were involved in 3 Loops, as shown in Pymol. The Loop 3, residues 122 to 127 turn into the α-helix structure (Figures 4D, G) and in Loop 1 residues 12 to 19 change into the β-folding structure (Figures 4F, I). Especially, in Loop 2 amino acid residues 68 to 85 turn into a combined structure of α-helix and β-folding (Figures 4E, H). From the above changes, it is clear that the presence of Mn^2+^ in the system can interact with these parts and lead to conformation changes in the Xylanase. According to the results of RMSD portion and structure changes, it could be seen that the presence of Mn^2+^ can increase the number of α-helix and β-folding in protein conformation. And it also increases the rigid structure of Xylanase, leading to the formation of binding sites difficult to reduce the catalytic activity of Xylanase. By analyzing the simulation data such as RMSD, we may discover that Mn^2+^ could play a role to help stabilize the conformation, which may further improve the hydrolysis ability of Xylanase. Therefore, Mn^2+^ could improve the ability of Xylanase to hydrolyze wheat bran, and the addition of Mn^2+^ resulted in catalytic activity changes, which could further potentially be applied in feruloyl oligosaccharides industrial application.

## 5 Conclusion

In this study, Xylanase hydrolyzed feruloyl oligosaccharides in wheat bran were studied Xylanase with a concentration of 4 mM of Mn^2+^, which could yield a highest product. In order to understand particular conformations of the subunit with the states of the binding change mechanism, MD were studied in this research. Subsequently, it was further clarified by MD that the enzyme underwent a conformational change in the system containing Mn^2^⁺, the presence of Mn^2+^ could expand the binding pocket and led to structural changes in the protein and resulted in an increase in the stability of the complex. This may provide some evidence that Mn^2+^ can increase the enzymatic activity of Xylanase in the hydrolysis of feruloyl oligosaccharides in wheat bran.

## Data Availability

The original contributions presented in the study are included in the article/supplementary material, further inquiries can be directed to the corresponding authors.
